# Primary Bladder Non-Hodgkin Lymphoma: A Case Report

**DOI:** 10.7759/cureus.52688

**Published:** 2024-01-21

**Authors:** Haobo Zheng, Hao Du, Junjiang Liu

**Affiliations:** 1 Urology, Hebei General Hospital, Shijiazhuang City, CHN

**Keywords:** diagnosis, case report, non-hodgkin lymphoma, tumor, bladder

## Abstract

Primary bladder lymphoma, a rare form of non-Hodgkin’s lymphoma, is diagnosed through histopathology and immunostaining. Most bladder lymphomas are of the B-cell type, with a higher incidence in women and often presenting with hematuria. This report details an exceptionally rare case of primary bladder T-cell lymphoma. A 50-year-old male, without hematuria or other symptoms, was diagnosed during a routine ultrasound. A computed tomography scan showed a tumor located in the anterior, right, and posterior walls. The patient underwent transurethral resection of the bladder lesion. Pathological examination of the tumor showed that it was composed of lymphoid tissue, in accordance with peripheral T-cell lymphoma of non-Hodgkin subtype.

## Introduction

Lymphoma is a type of cancer that originates in the lymphatic system. It manifests as the abnormal growth of lymphocytes. Lymphomas are broadly categorized into two main types: Hodgkin lymphoma (HL) and non-Hodgkin lymphoma (NHL). Diagnosis involves various tests, including biopsies and imaging. Lymphocytes can circulate throughout the bloodstream and lymph nodes, so lymphoma may present as tumors or abnormal cell growths in organs such as the spleen, bone marrow, or even the gastrointestinal and urological tract [1,2].

However, regarding the bladder, the primary NHL of the bladder is extremely rare and represents less than 0.2% of extra-nodal lymphomas [3,4], due to a lack of lymphoid tissue in the bladder. The low incidence makes both diagnosis and therapy challenging. Moreover, treatment is not well-defined, and the prognosis remains unknown. A case report on bladder lymphoma could offer valuable insights to researchers and clinicians. More available data can contribute to optimizing therapeutic strategies for bladder lymphoma. Thus, we report a case of primary NHL of the bladder while reviewing the appropriate literature.

## Case presentation

**Figure 1:**
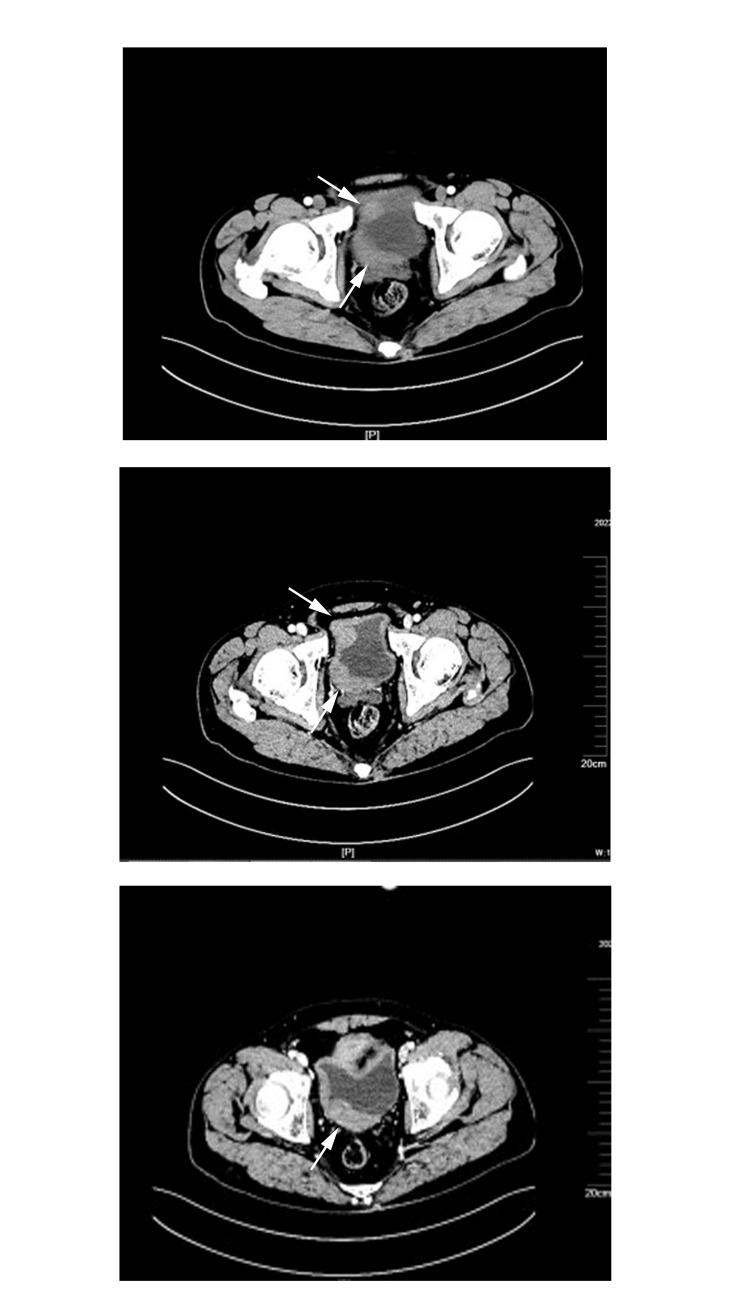
Representative CT of the bladder tumor The pelvic CT scan shows uneven thickening of the anterior wall, right wall, and posterior wall of the bladder (indicated by the arrows).

A 50-year-old man was admitted to the hospital after a bladder mass was discovered by ultrasound during a regular annual examination. This patient did not exhibit hematuria or other symptoms. He had a history of cerebral hemorrhage and hypertension, whereas he had no history of surgery in the past. Physical examination and laboratory tests (blood and urine) showed no significant abnormalities. There were no enlarged lymph nodes or palpable liver or spleen upon physical examination. The ultrasound examination of the urinary system during the regular annual examination revealed a low-echoic mass on the posterior wall of the bladder. A pelvic computed tomography (CT) scan showed that the bladder was adequately filled, with uneven thickening of the anterior wall, right wall, and posterior wall, particularly pronounced in the posterior wall with a thickness of about 25 mm. Enhanced scanning showed relatively uniform enhancement, with average CT values of approximately 35 Hounsfield Units (HU), 45 HU, 63 HU, and 72 HU in the pre-contrast, arterial, venous, and equilibrium phases, respectively (Figure 1). The lesion had an unclear boundary with the prostate, and the bladder wall near the right ureteral orifice appeared slightly thicker.

**Figure 2:**
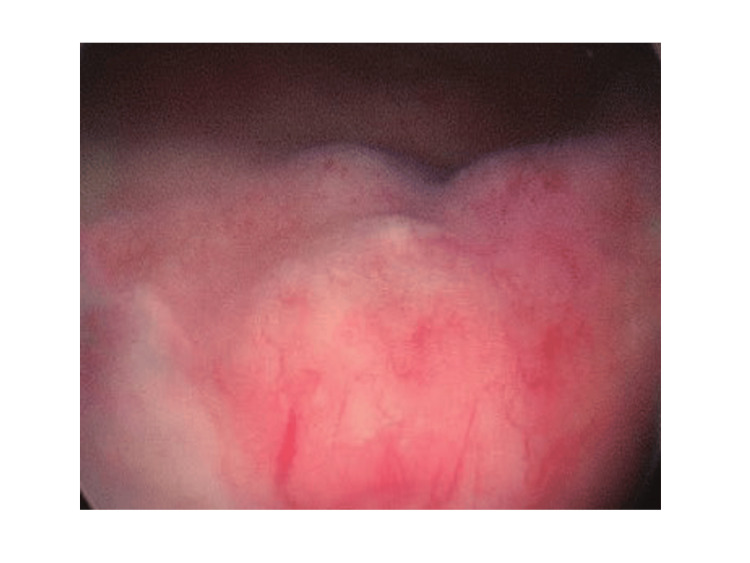
Intraoperative endoscopic figure of the bladder tumor Intraoperatively, the bladder wall is observed to have diffuse thickening, a friable texture, and be prone to bleeding.

Then, the patient underwent transurethral resection of the bladder lesion under general anesthesia. Intraoperatively, it was observed that the bladder had good capacity, and the bilateral ureteral orifices had a fissure-like appearance with normal peristalsis and urine flow (Figure 2). Extensive thickening of the bladder wall was observed on the right side of the bladder base and right lateral wall, with a smooth surface and friable texture, which bled easily upon touch.

**Figure 3:**
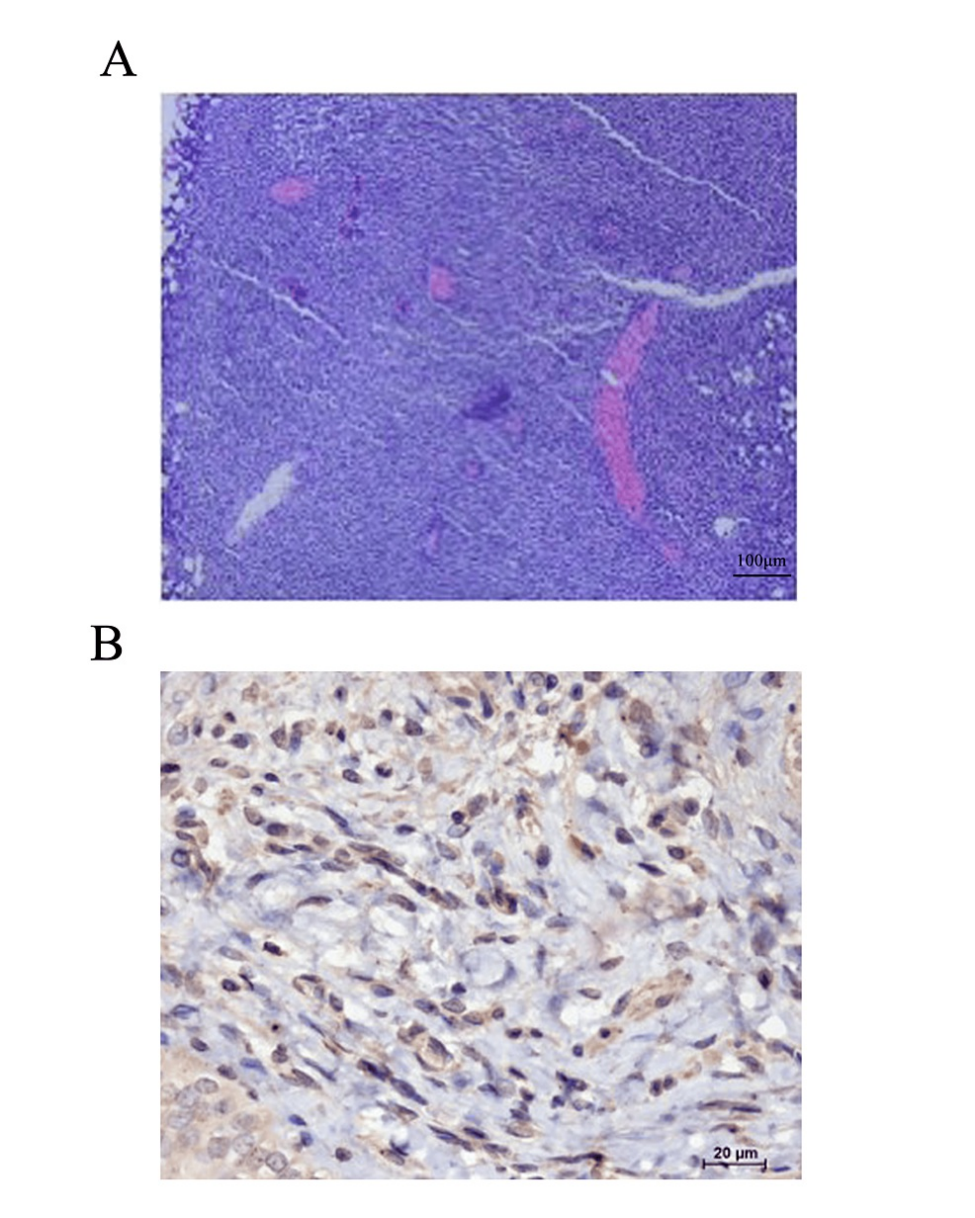
Representative histological and immunohistochemical figures of the bladder tumor (A) Histological examination (HE ×100) revealed the presence of dense lymphocytic infiltration and some lymphoid follicles. Small to medium-sized centrocyte-like lymphocytes form diffuse infiltrates within the bladder lymphoid epithelial lesion (scale bar:100μm). (B) Immunohistochemistry examination reveals strong expression of CD3 in tumors (scale bar: 20μm).

Postoperative pathological examination of the bladder tumor showed (Figure 3) a proliferative lesion of lymphoid tissue in accordance with peripheral (cytotoxic) T-cell lymphoma, non-Hodgkin subtype (Figure 3A). Immunohistochemical staining (Figure 3B) indicated CD3 (+).

Therefore, the final diagnosis was non-Hodgkin peripheral T-cell lymphoma of the bladder. The patient was subjected to CHOP (cyclophosphamide, doxorubicin, vincristine, and prednisone) chemotherapy after surgery.

## Discussion

Bladder lymphoma is relatively rare, accounting for approximately 0.2% of primary tumor lesions and about 1.8% of secondary lesions [5]. Most lymphomas of the bladder are NHL of the B-cell type. Lontos et al. found that 60.3% of primary bladder lymphoma cases were diffuse large B-cell lymphoma while 22.7% of these cases were mucosa-associated lymphoid tissue (MALT) lymphoma [6]. For example, Nerli et al. reported a case of primary B-cell NHL of the bladder in a 69-years-old male [7] while Díaz-Peromingo et al. documented a case of primary bladder NHL in a 79-year-old Caucasian man, characterized by large B-cell lymphoma [8]. Bacalja reported a case of primary mucosa-associated lymphoid tissue (MALT) lymphoma of the bladder [9]. Nevertheless, we presented a case of primary bladder NHL characterized by T cells, which is a less common subtype of primary bladder NHL. Our search of the English-language medical literature yielded only approximately five previously reported cases of primary bladder T-cell lymphoma [10-13].

The cause of primary NHL in the bladder remains unclear due to its rarity. Some authors suggest that lymphoma in this context might be linked to chronic cystitis, with tumors potentially arising from the lymphocytes in the bladder's submucosal layer as a response to inflammation. Another theory put forth by other authors suggests the possibility of residual embryonic cloaca leading to lymphoid proliferation in adulthood [14].

Bladder lymphoma is more common in adults, with a higher incidence in females than males, with a male-to-female ratio of 1:6.5 [15]. The median age of the patients at diagnosis is 64 years (range 20-85) [16]. Clinical symptoms typically include painless gross hematuria, which may be accompanied by difficulty urinating, urinary frequency, urgency, and dysuria. In our case, the patient did not exhibit any symptoms; instead, the condition was discovered during a routine ultrasound examination. In contrast, hematuria was reported in four other cases of primary bladder T-cell lymphoma [10-13]. However, during the transurethral resection surgery, we observed that the tumor tissue bled easily upon contact with surgical instruments. Based on this, we speculate that the absence of hematuria symptoms in the patient of this case might be due to the disease being in an early stage, where the tumor tissue has not yet damaged the bladder mucosa.

Diagnostic imaging, such as CT scans and MRI, often do not provide substantial additional information for diagnosis, as the findings closely resemble those of transitional cell carcinoma. Nevertheless, there are specific characteristics that are more frequently associated with lymphomas, including diffuse infiltration, and less frequent retraction of the urinary bladder wall [17]. For example, on CT scans, bladder lymphoma appears as diffuse thickening of the bladder wall, with a soft tissue density on plain scans and uniform, moderate to marked enhancement on contrast-enhanced scans [7,8]. In this case, the CT findings are typical, with extensive thickening observed on the right side of the bladder base and the right bladder wall, consistent with the literature. MRI examinations show low or iso-low signal on T1-weighted imaging, high or slightly high signal on T2-weighted imaging, and progressive delayed enhancement in the third phase [17]. Imaging studies provide valuable references for lesion detection while pathological tissue examination and immunohistochemical staining, including CD2, CD3, CD4, CD5, CD8, CD43, and CD56, can further clarify the nature and origin of the tissue [18]. In this case, immunohistochemical staining showed CD3 (+).

The treatment of bladder lymphoma primarily aims to control the progression of local lesions. Various therapeutic approaches are accessible for primary NHL, including transurethral resection of bladder tumors, partial cystectomy, radical cystectomy, radiotherapy, and chemotherapy. The choice of treatment depends on factors such as the tumor's clinical behavior, risk of disease progression, the patient's life expectancy, and overall health. Currently, chemotherapy is the preferred treatment option because of its effectiveness in addressing early systemic diseases that may not have been detected. Regarding the choice of chemotherapy, various regimens including R-CHOP, CHOP, ChlVP, and ChlD have proven effective in the treatment of low-grade bladder lymphomas. Among these, R-CHOP is the most commonly reported regimen in the literature [5,9]. In a case of primary bladder B-cell lymphoma, the patient received chemotherapy (6 cycles of R-CHOP) and achieved a complete response [15]. Another patient with primary bladder lymphoma (diffuse large B-cell type) underwent chemotherapy (6 cycles of CHOP) and radiotherapy, achieving complete remission [4]. Additionally, a primary bladder MALT lymphoma received R-CHOP chemotherapy and radiotherapy, resulting in radiological remission but with metabolic tumor progression [9].

## Conclusions

In summary, bladder lymphoma is a rare malignant tumor with nonspecific symptoms. Early confirmation through pathological tissue examination is crucial for early detection, diagnosis, and treatment, which can ultimately extend the patient's survival period.
